# Gait pattern can alter aesthetic visual impression from a third-person perspective

**DOI:** 10.1038/s41598-024-56318-5

**Published:** 2024-03-19

**Authors:** Sakiko Saito, Momoka Saito, Megumi Kondo, Yoshiyuki Kobayashi

**Affiliations:** 1 Liberal Arts and Sciences, Nippon Institute of Technology, Saitama, Japan; 2 Faculty of Human Life and Environmental Sciences, Ochanomizu University, Tokyo, Japan; 3 Human Augmentation Research Center, National Institute of Advanced Industrial Science and Technology, Chiba, Japan; 4 Present Address: Department of Transdisciplinary Science and Engineering, School of Environment and Society, Tokyo Institute of Technology, Tokyo, Japan; 5 Present Address: Faculty of Core Research, Natural Sciences Division, Ochanomizu University, Tokyo, Japan

**Keywords:** Psychology, Health care

## Abstract

Beauty is related to our lives in various ways and examining it from an interdisciplinary approach is essential. People are very concerned with their appearance. A widely accepted beauty ideal is that the thinner an individual is, the more beautiful they are. However, the effect of continuous motion on body form aesthetics is unclear. Additionally, an upright pelvic posture in the sagittal plane during walking seems to affect the aesthetic judgments of female appearance. We directly analyzed the influence of body form and walking pattern on aesthetic visual impressions from a third-person perspective with a two-way analysis of variance. Captured motion data for three conditions—upright pelvis, normal pelvis, and posteriorly tilted pelvic posture—were applied to each of three mannequins, representing thin, standard, and obese body forms. When participants watched stimulus videos of the mannequins walking with various postures, a significantly higher score for aesthetic visual impression was noted for an upright pelvic posture than for a posteriorly tilted pelvic posture, irrespective of body form (*F*_(2, 119)_ = 79.89, *p* < 0.001, *η*^2^ = 0.54). These findings show that the third-person perspective of beauty can be improved even without being thin by walking with an upright pelvic posture.

## Introduction

Beauty is an essential concept that is related to our lives in various ways; thus, it needs an interdisciplinary approach^1^. There is ample evidence that people are concerned with their own appearance^2^. Several social and psychological studies have demonstrated that one’s appearance is important for evolutionary reasons, such as mate selection^3^ as well as for social aspects^4^. Those that are more attractive than others are often given preferential treatment in employment opportunities^5–7^, educational institutions^8^, and judicial decisions^9^.

Body form is one of the main sources for one’s attractiveness rating. A widely accepted ideal is that the thinner individual is, the more beautiful they are^10–16^. Internalization of such ideals has a strong influence on body satisfaction of individuals and could be a critical risk factor for disordered eating behaviors^10,13–16^. According to the World Health Organization, as of 2019, 14 million people have experienced eating disorders, including nearly 5.2 million adolescents and young adults (10–24-years-old). Women are twice as likely as men to experience these disorders^17^. Thin-ideal body form appears to be influenced by sociocultural factors, such as mass media exposure^10,13,14^. The recent proliferation of social networking services (SNSs) is likely to further strengthen the current trends^18^. Is there no way to improve impressions of beauty from a third-person perspective other than by being thin?

Recently, aesthetic visual impression from a third-person perspective has been linked to body form and motion^19,20^. For example, Cazzato et al.^19^ investigated the effects of implied motion and body size of a virtually rendered human body model—created using static images of the same model—on aesthetic evaluation by a third person and reported that both elements significantly affected aesthetic evaluation, with a preference for the stimulus of more dynamic and thinner models. Moreover, they revealed that implied motion has a greater effect than body weight for the beauty judgments of posture. Similarly, Sugawara et al.^20^ investigated the effects of posture and body size on the aesthetic evaluation by a third person using static images. They reported that both elements significantly affected aesthetic evaluation, with a preference for images presenting an upright posture regardless of body size.

These findings imply that appropriate motion improves beauty impressions in terms of a third person’s aesthetic visual impression, which may help individuals experiencing unhealthy eating behaviors owing to beauty-related reasons. However, previous studies have used static images to evaluate aesthetic visual impressions from a third-person perspective. In the real world, aesthetic visual impressions are created through continuous motion^21–23^. Furthermore, currently, several young people share videos with continuous motion on SNSs. Therefore, understanding how aesthetic visual impressions from a third-person perspective are linked to body form and continuous motion is essential.

In this study, out of the various human motions that possibly influence aesthetic visual impression from a third-person perspective, we focused on walking. Walking is among the most essential means of human locomotion and is one of the major themes posted on SNSs. In March 2023 (i.e., at the time of submitting this paper), the hashtag “walking” had 28 million posts on Instagram, and the hashtag “walk” had 36 million posts on Instagram. Walking is a movement that attracts a lot of attention on SNS, but we usually walk unconsciously. Additionally, gait kinematics is associated with female attractiveness^4,24–28^. Several researchers have revealed the biomechanical factors of female gait attractiveness from the observers’ viewpoint, suggesting that upright pelvic posture in the sagittal plane is vital in the aesthetic judgments of female appearance^4,26–28^. This tendency is also observed in the walking posture of experienced ballet dancers^28^ and professional fashion models^4,26^ and the static posture of Latin American-style professional dancers^29^.

Here, we investigate the contribution of body forms and walking patterns to aesthetic perception of female appearance by using continuous motion videos. Considering previous studies^19,20^ that revealed the effect on aesthetic impression of postural changes was greater than that of body form, we hypothesized that the aesthetic visual impression score would be higher for an obese mannequin walk with an upright pelvic posture than for a thin mannequin walk with a posteriorly tilted pelvic posture. This study aimed to determine the influence of body forms and walking patterns on a third person’s aesthetic visual impression regarding a walking individual. To this end, stimulus videos that included stimuli that controlled the body form factor and walking pattern factor independently, were played to enable the observer to rate the aesthetic visual impression. As previous studies used still static images, this novel study used continuous motion to examine the effects of both body forms and walking patterns on aesthetic impressions. By using continuous motion videos, we highlight that it is necessary to verify impressions from one’s appearance, including motion. The model in the video stimulus was asked to walk with three pelvic postures (upright pelvis, normal pelvis, and posteriorly tilted pelvic posture); we measured gait using a motion capture system. We applied the captured motion data to the body-form-adjustable digital mannequin and rendered three walking videos with thin, standard, and obese body forms.

## Results

**Figure 1:**
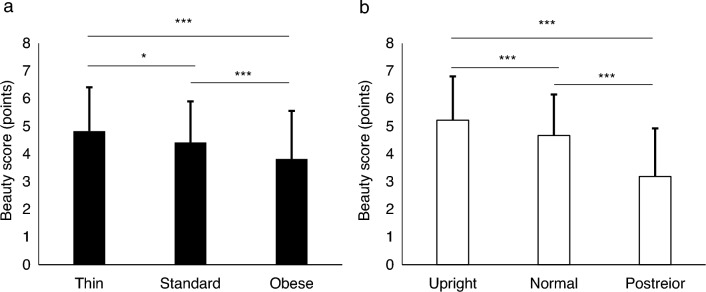
Mean and standard deviation of beauty subjective ratings for each body form (**a**) and walking pattern (**b**). Error bars indicate standard deviation. *p < 0.05, ***p < 0.001.

We performed a two-way analysis of variance (ANOVA) to investigate the effect of two factors, body form and walking pattern directly. As Mauchly’s sphericity test was significant (body form: *p* < 0.001, walking pattern: *p* = 0.01, interaction between two factors: *p* < 0.001), we used the degrees of freedom adjusted by Green-House-Gazer-Epsilon. The two-way ANOVA revealed that the main effects of body form [*F*_(2, 111)_ = 22.85, *p* < 0.001,* η*^2^ = 0.25 (corresponding to a large effect size)] and walking patterns [*F*_(2, 119)_ = 79.89, *p* < 0.001, *η*^2^ = 0.54 (corresponding to a large effect size)] on the stimulus videos’ aesthetic visual impression score were both significant, while the interaction between body form and walking pattern was not significant [*F*_(3, 219)_ = 1.46, *p* = 0.22, *η*^2^ = 0.02 (corresponding to a negligible effect size)]. The post-hoc analysis revealed significant differences between various pairing combinations of body forms (Fig. 1a), as well as walking patterns (Fig. 1b). Table 1 presents the beauty scores for each stimulus video. The scores for the stimulus video of a thin mannequin walk were significantly higher than those for the stimulus videos of a standard (*p* = 0.01) and obese mannequin walk (*p* < 0.001). The scores for the stimulus video of a standard mannequin walk were significantly higher than those for the stimulus video of an obese mannequin walk (*p* < 0.001). Furthermore, the scores for the stimulus videos of a mannequin walk with an upright pelvic posture were significantly higher than those for the stimulus videos of a mannequin walk with a normal pelvis (*p* < 0.001) and a posteriorly tilted pelvic posture (*p* < 0.001); the scores for the stimulus videos of a mannequin walk with a normal pelvis were significantly higher than those for the stimulus videos of a mannequin walk with a posteriorly tilted pelvis (*p* < 0.001).

**Table 1:** Descriptive statistics of beauty subjective ratings.

	Thin	Standard	Obese
Upright	5.78 ± 1.34	5.26 ± 1.25	4.62 ± 1.64
	Range: 2–7	Range: 2–7	Range: 1–7
Normal	5.25 ± 1.47	4.79 ± 1.59	3.97 ± 1.88
	Range: 1–7	Range: 1–7	Range: 1–7
Posterior	3.46 ± 1.89	3.24 ± 1.85	2.87 ± 1.52
	Range: 1–7	Range: 1–7	Range: 1–7

Continuous values are presented as the mean ± standard deviation, and a lower row are presented as the range of scores.

## Discussion

This study investigated how a third person’s aesthetic visual impression on walking is influenced by body forms and walking patterns. We directly analyzed the influence of body forms and walking patterns on the aesthetic perception by using a two-way ANOVA. Although there was no significant interaction between body form and gait pattern, when participants watched the stimulus video of a mannequin walking with different postures, a significantly higher score was observed for a thinner than for an obese mannequin. Moreover, a significantly higher aesthetic visual impression score was noted for an upright pelvic posture than for a posteriorly tilted pelvic posture. When participants watched the stimulus videos of the mannequin walk, significantly higher scores for aesthetic visual impression were noted for an upright pelvis than for a posteriorly tilted pelvic posture, irrespective of body form. The latter result confirms our initial hypothesis and indicates that one can improve their beauty impressions from a third-person perspective, even if one is not thin, by walking with an upright pelvic posture.

We ascertained the success of our critical body form manipulation and the influence movement on body aesthetic impression. Our results are consistent with those of Fan et al.^3^, Wang et al.^15^, and Cazzato et al.^19^, who found that thinner body is a dominant determinant of female physical attractiveness. In addition, the movement of pelvis during walking is also a reasonable way to change female physical attractiveness while not being affected by the walker’s body form. Therefore, beyond static image, analyzing the impression of a person’s appearance in combination with dynamic elements could change previous observers’ perception of body attractiveness.

The effects of changes in walking pattern on the aesthetic impression did not differ between body forms. Considering previous studies^19,20^, it was quite possible that an interaction between body form and gait pattern would be significant, but this was not the case. The walking motion taken from one standard body-typed female was expressed using different body forms. It is unclear whether an obese person or a slim person would be able to reproduce the same motion when tilting their pelvis. For example, decrease in body weight changes the kinematic characteristics of each joint during human walking^30,31^. The method of this study was useful for systematically changing body forms and walking patterns. Additionally, by using the walking movements of multiple people with different body types, results that support this hypothesis could be obtained.

The mass media has a great influence on the sociocultural aspects of body aesthetics. The mass media that makes thinness desirable has a negative impact on the three factors of women who see and hear about it: dissatisfaction with their own body form, internalization of the ideal of thinness, and beliefs about eating behavior^32^. Major companies in the fashion industry issued a statement prohibiting the use of models that are too thin in 2017^33^. Although the broader use of SNSs has accelerated negative effects on body images^13^, we can use SNSs to help build a healthy body image. SNSs allows us to easily exchange short videos. As most people consider exercise to be an important component of health^19^, the current results, which focused on the dynamic elements of body appearance, could promote a healthy body image and improve eating habits.

Pelvic alignment is known to affect whole-body posture, and altering whole-body posture may have affected the third person’s aesthetic visual impression. For example, Day et al.^34^ reported that the voluntary alteration of pelvic posture in the sagittal plane affected the lumbar lordotic curve, hip position, and head position during quiet standing. Kobayashi et al.^27^ and Saito et al.^28^ reported that during walking, an upright pelvic posture is linked to hip flexion, trunk extension, and a greater range of pelvic and ankle motion, whereas a posteriorly tilted pelvic posture is linked to hip extension, trunk flexion, and a smaller range of pelvic and ankle motion. An upright pelvic posture and the associated hip flexion, trunk extension, and a greater range of pelvic and ankle motion will lead to an upright posture and more dynamic motion during walking. Such features align with those of still images that have been perceived as more aesthetic impression by a third person in previous studies^19,20^. Therefore, it is reasonable to infer that the aesthetic visual impressions evaluated by the participants in the current study were based on whole-body posture affected by pelvic alignment, which was voluntarily adjusted by the model.

Walking while maintaining an appropriate pelvic angle may help those who are struggling to be thin for the sake of beauty. In this study, the model in the stimulus video was instructed to walk with different pelvic alignments. After a short practice period, she was able to walk on a treadmill with a different pelvic alignment and successfully alter a third person’s aesthetic visual impression. This result suggests that individuals can improve a third person’s aesthetic visual impression relatively easily by adjusting their pelvic alignment. However, an excessively tilted pelvic posture in the anterior direction may induce low back dysfunction, such as lumbar lordosis^34^. Lews et al.^35^ showed that excessive forward tilting of the pelvis is undesirable from an aesthetic point of view, too, but the optimal angle was unclear. When changing one’s walking pattern, the effectiveness varies depending on the intervention method, and it is also difficult to change and maintain a new walking pattern^36,37^. Therefore, the appropriate angle of pelvic tilting in sagittal plane and intervention method should be examined in future studies.

Markerless motion capture systems for motion analysis are inexpensive and requires less time and labor, but there are issues with accuracy. Kinect, which is one of the systems, has the validity of gait analysis in comparison to a marker-based motion capture system, when used properly^38^. Kinect and iPi Recorder systems has been proven to be useful by using two or more cameras to acquire data^39–41^ and by specifying the motion plane (i.e., sagittal plane) to be analyzed^42,43^. When rendering the digital mannequin, we attempted to reproduce motion more accurately by removing motion artifacts^44^ by using two cameras. Furthermore, we used the digital mannequin's gait seen from the left side as a stimulus video.

Certain limitations must be acknowledged when interpreting the current results. The findings were based on a limited number of videos provided to participants. Stimulus videos were based on the walking data of a single model using software that could generate a body-form-adjustable digital mannequin. The videos’ viewpoint solely pertained to the sagittal plane. Different models have different proportions, such as leg length, and different software packages generate different body forms. Different viewpoints (e.g., frontal plane) may result in participants focusing on different body parts. Different typed digital mannequin could affect the impression of observers^45^. Additionally, our participants were only young Japanese adults. People’s impressions of body attractiveness could differ depending on the region and cultural background of the observer^46^. Therefore, further research is needed to deepen our understanding of walking patterns that can improve beauty in terms of a third person’s aesthetic visual impressions.

In conclusion, when participants watched the stimulus videos of the mannequin walking with various postures, we found a significantly higher score for aesthetic visual impression for an upright pelvic posture than for a posteriorly tilted pelvic posture, irrespective of body form. Therefore, our findings indicate that beauty can be improved in terms of a third person’s aesthetic visual impressions—even if one does not become thin—by walking with an upright pelvic posture. Only the anteroposterior tilted angle of the pelvis was manipulated. The upright pelvic posture is a common element in other aesthetic physical activities such as dance^28,29^, and our results contribute to expanding the understanding of body aesthetics. In future research, we will consider how other parts of the body change as the pelvis tilts, and this will provide fundamental knowledge for considering what approach is best when changing movement. Furthermore, by using a method in which the observers create the walking motion themselves, we can more accurately reproduce the walking motion that the observer envisions.

## Materials and methods

### Participants

Sixty-eight young people aged 18–28 years (mean = 21.2 ± 2.1 years; 35 women and 33 men) volunteered to participate. Participants were recruited mostly from the student population at Ochanomizu University and Nippon Institute of Technology in Japan. None of the participants reported any current neurological or psychiatric disorder. This study was conducted in accordance with the World Medical Association’s Declaration of Helsinki. All study protocols were submitted and reviewed by the local institutional review board (Committee on Ergonomic Experiments of the National Institute of Advanced Industrial Science and Technology) prior to starting the experiment. The board concluded that the study protocols were exempted from full review, in accordance with the regulations of the Institute (HF2021-1165). All participants gave written informed consent before participating.

### Stimulus videos

The whole-body three-dimensional trajectory of a young female model wearing sneakers and walking on a treadmill at a comfortable self-selected speed was recorded using an Xbox One Kinect sensor (Microsoft, Redmond, WA, USA) and iPi Recorder software (iPi Soft, Moscow, Russia). Two Kinect sensors were placed orthogonally—one on the side and one behind the treadmill. The model was instructed to walk with three pelvic postures: (1) a normal pelvic condition in which they were instructed to walk as casually as possible; (2) an upright pelvic condition in which they were asked to walk with a more upright pelvic posture than a normal pelvic posture; and (3) a posteriorly tilted pelvic condition in which they were asked to walk with a posteriorly tilted pelvic posture than a normal pelvic posture. The model was aware of the study’s purpose. Other body parts’ (lower limbs, trunk, etc.) movements were not specifically dictated, thereby leaving them influenced by pelvic posture. After sufficient practice time to become accustomed to walking on the treadmill under each condition, two sets of 1-min-long walking data were recorded for each condition. Two researchers of biomechanics visually checked whether the differences owing to conditions were reflected in the model’s walking and whether the motion was consistently reproduced in two tests.

**Figure 2:**
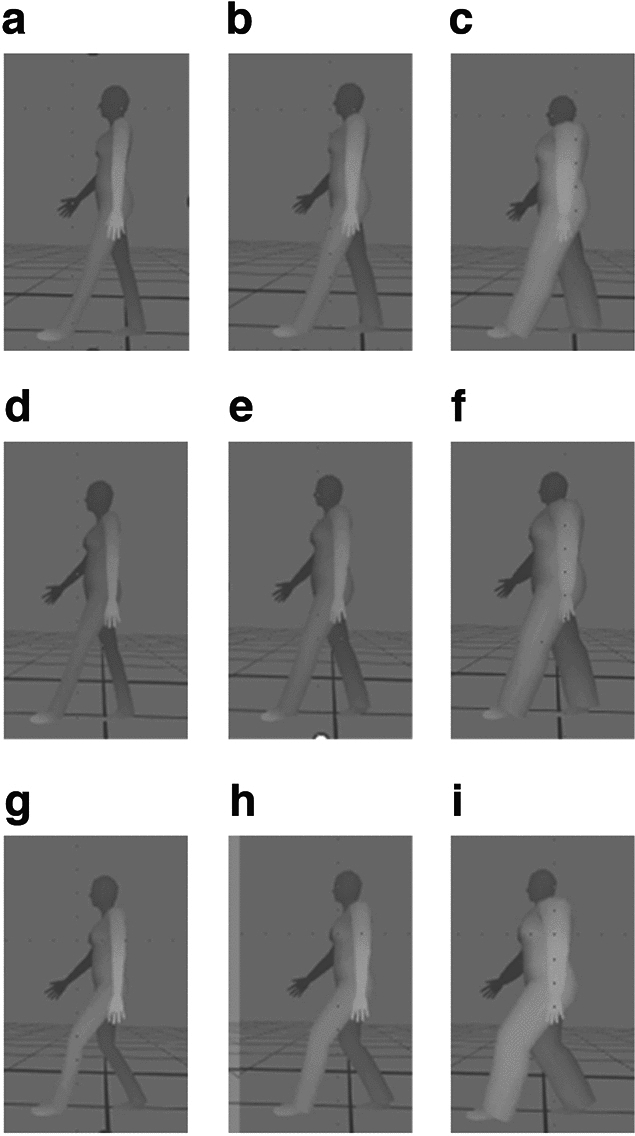
Human walking mannequins created using motion capture system (iPi Mocap Studio (iPi Soft, Moscow, Russia)). From left to right, the mannequins depict the body form variations in the same walking patterns. From top to bottom, the pelvic angle changes from upright to posterior tilt in the same body forms: (**a**) a thin mannequin with an upright pelvic posture, (**b**) a standard mannequin with an upright pelvic posture, (**c**) an obese mannequin with an upright pelvic posture, (**d**) a thin mannequin with a normal pelvic posture, (**e**) a standard mannequin with a normal pelvic posture, (**f**) an obese mannequin with a normal pelvic posture, (**g**) a thin mannequin with a posterior pelvic posture, (**h**) a standard mannequin with a posterior pelvic posture, (**i**) an obese mannequin with a posterior pelvic posture.

IPi Mocap Studio (iPi Soft, Moscow, Russia) was used to render the body-form-adjustable digital mannequin. To clean up the gaps between the tracking data and the digital mannequin, we manually edited the tracking results using iPi Mocap Studio's editing tools. Then two filters were used on the tracked skeleton: Jitter removal filter and trajectory filter. Jitter removal filter removed unexpected noise and the trajectory filter removed small noises that remain after applying the jitter removal filter. At first, the mannequin’s standard body form was rendered by inputting the model’s biological sex (i.e., female) and height (1.58 m). By adjusting the body mass index tab in the software program, we rendered thin, standard, and obese mannequins. Captured motion data for the three conditions (upright pelvis, normal pelvis, and posteriorly tilted pelvic posture) were applied to each of the three mannequins (thin, standard, and obese). Thus, nine sets of three-dimensional motion data were generated. Figure 2 presents an image from each video. As the values in the sagittal plane captured by Kinect sensor are more accurate^42,43^, the walking motion with rendered digital mannequin was displayed in the sagittal plane.

### Third person’s subjective evaluation

A questionnaire for participants to score their aesthetic visual impression of each stimulus video was created using Microsoft Forms (Microsoft, Redmond, WA, USA). The questionnaire comprised the following two components: (1) demographic and (2) experimental questions. In the first component, participants were asked to input their age and sex. In the second component, participants were asked to score their aesthetic visual impression—in terms of “is beautiful (in Japanese, *kireidearu*)”—of the stimulus videos using a seven-point scale (1 = not at all, 7 = very much so). Previous studies have used similar questionnaires to evaluate the attractiveness of the human movements and body forms^4,26,47^. Each participant assessed nine stimuli (three body forms and three gait patterns) presented in random order (Video 1-9).

### Statistical analysis

To ascertain how a third person’s aesthetic visual impression is associated with body forms and walking patterns, we used a two-way ANOVA with repeated measures. As there was no applicable multiple comparison method for ANOVA with repeated measures, the *Bonferroni* method was used for post-hoc analysis. This method has a feature that as the number of groups increases, the risk of Type II error increases. Each factor had three groups, resulting in an alpha error probability of 0.049, which was sets lightly less than the significance threshold of 0.050. The significance threshold was set at *p* < 0.05. All statistical analyses were performed using SPSS statistical software package (IBM SPSS Statistics, version 21; SPSS Inc., Chicago, IL, USA).

### Supplementary Information

Supplementary Video 1.

Supplementary Video 2.

Supplementary Video 3.

Supplementary Video 4.

Supplementary Video 5.

Supplementary Video 6.

Supplementary Video 7.

Supplementary Video 8.

Supplementary Video 9.

## Data Availability

The datasets used and/or analyzed during the current study are available from the corresponding author on reasonable request.
